# Treatment with a combination of the ErbB (HER) family blocker afatinib and the IGF-IR inhibitor, NVP-AEW541 induces synergistic growth inhibition of human pancreatic cancer cells

**DOI:** 10.1186/1471-2407-13-41

**Published:** 2013-01-31

**Authors:** Nikolaos Ioannou, Alan M Seddon, Angus Dalgleish, David Mackintosh, Helmout Modjtahedi

**Affiliations:** 1 School of Life Sciences, Kingston University London, Kingston-upon-Thames, Surrey KT1 2EE, UK; 2 Department of Cellular and Molecular Medicine, St George’s University of London, London, UK

**Keywords:** EGFR, IGF-IR, Afatinib, NVP-AEW541, Pancreatic cancer

## Abstract

**Background:**

Aberrant expression and activation of the IGF-IR have been reported in a variety of human cancers and have been associated with resistance to HER targeted therapy. In this study, we investigated the effect of simultaneous targeting of IGF-IR and HER (erbB) family, with NVP-AEW541 and afatinib, on proliferation of pancreatic cancer cells.

**Methods:**

The sensitivity of a panel of human pancreatic cancer cell lines to treatment with NVP-AEW541 used alone or in combination with afatinib, anti-EGFR antibody ICR62, and cytotoxic agents was determined using the Sulforhodamine B colorimetric assay. Growth factor receptor expression, cell-cycle distribution and cell signalling were determined using flow cytometry and western blot analysis.

**Results:**

All pancreatic cancer cell lines were found to be IGF-IR positive and NVP-AEW541 treatment inhibited the growth of the pancreatic cancer cell lines with IC50 values ranging from 342 nM (FA6) to 2.73 μM (PT45). Interestingly, of the various combinations examined, treatment with a combination of NVP-AEW541 and afatinib was superior in inducing synergistic growth inhibition of the majority of pancreatic cancer cells.

**Conclusion:**

Our results indicate that co-targeting of the erbB (HER) family and IGF-IR, with a combination of afatinib and NVP-AEW541, is superior to treatment with a single agent and encourages further investigation *in vivo* on their therapeutic potential in IGF-IR and HER positive pancreatic cancers.

## Background

Despite major advances in cancer diagnosis and therapy in the last few decades, pancreatic cancer remains one of the most fatal types of human cancer with the mean survival rate of less than 6 months [1,2]. In 2012, pancreatic cancer is estimated to be the ninth most commonly diagnosed cancer (43,920) but the fourth leading cause of cancer deaths (37,390) after lung, colorectal and breast cancers in the USA [3]. Worldwide, pancreatic cancer was responsible for an estimated 266,000 deaths in 2008 [4].

Since the early 1980s, aberrant expression and activation of Receptor Tyrosine Kinases (RTKs) such as the ErbB (HER) family of receptors have been shown to be implicated in several human malignancies and in some cases have been associated with a poor prognosis [5-8]. The ErbB (also called HER or EGFR) family of receptors is one of the best characterized RTK and consists of four family members namely; EGFR (HER-1), ErbB2 (HER-2), ErbB3 (HER-3) and ErbB4 (HER-4) [9,10]. Activation of the HER family members following ligand binding, leads to the activation of several downstream signalling pathways including the Ras-Raf-mitogen activated protein kinase (MAPK), phosphatidylinositol 3 kinase protein (PI3K)/AKT pathway, PLC- γ-protein kinase C (PKC) and signal transducers and activators of transcription (STAT) pathway. Deregulation of the HER family pathway can result in increased cell proliferation, motility, evasion of apoptosis and angiogenesis and these are some of the hallmarks of human cancers [9,11,12]. To date, several HER targeting agents have been approved for treatment of human cancers including metastatic colorectal cancer [anti-EGFR monoclonal antibodies (mAbs) cetuximab and panitumumab], non-small cell lung cancer [tyrosine kinase inhibitors (TKIs) gefitinib and erlotinib] ,early stage and metastatic breast (anti-HER-2 mAbs trastuzumab and pertuzumab, and dual EGFR/HER2 TKI lapatinib), head and neck (cetuximab), metastatic stomach cancers (trastuzumab) and pancreatic (erlotinib). However, despite these advances, many patients simply do not respond to or acquire resistance to therapy with the HER inhibitors [8].

The Insulin-like Growth Factor receptor (IGF-IR) is another very well characterized RTK and the main mediator of the biological action of IGF-I and IGF-II [13,14]. The IGF signalling network includes the IGF-I and IGF-II ligands, insulin, the cell surface receptors IGF-IR, IGF-IIR and the Insulin receptor (IR) as well as a group of regulatory IGF binding proteins (IGFBPs) [14-16]. The IGF-IR signalling axis is implicated in the regulation of a number of cellular processes including cell growth, survival and cell differentiation, and its aberrant activation has been associated with increased cell proliferation, reduced apoptosis, transformation, angiogenesis and increased cell motility and resistance to chemotherapy and radiotherapy in several types of human cancers [14,17,18]. As a result, the IGF-IR network has emerged as an attractive target for the development of new therapeutic strategies and a number of small molecule IGF-IR TKIs and anti-IGF-IR mAbs have been developed which are at different stages of preclinical evaluations and clinical trials in several types of human malignancies. In addition, recent studies have demonstrated that IGF-IR is implicated in resistance to anti-HER targeted therapy and consequently, simultaneous targeting of HER family members and IGF-IR may lead to a superior therapeutic effect in cancer patients.

We have recently reported the superiority of afatinib, an irreversible erbB family blocker, compared to the anti HER monoclonal antibody (mAb) ICR62 and first generation TKI erlotinib in inhibiting the growth of a panel of human pancreatic tumour cells [19]. The aim of this study was to investigate the sensitivity of the same panel of pancreatic cancer cell lines to treatment with an IGF-IR TKI, NVP-AEW541 [20], when used alone or in combination with afatinib, anti-EGFR mAb ICR62 or gemcitabine. In addition, we investigated the effect of these inhibitors on the phosphorylation of HER receptors, IGF-IR and downstream molecules such as MAPK and AKT and whether there was any association between the expression of the receptor and sensitivity to treatment.

## Methods

### Tumour cell lines

A panel of 7 human pancreatic cancer cell lines was used in this study including BxPC3, PT45, MiaPACA2, PANC-1, AsPc-1, Capan-1 and FA6 as well as control EGFR overexpressing head and neck cancer cell line HN5 and breast carcinoma cell line MCF-7. AsPc-1 and Capan-1 cell lines were kindly provided by Dr. Charlotte Edling (Blizard Institute of Cell and Molecular Science, Barts and The London School of Medicine and Dentistry). All cell lines were cultured routinely at 37°C in a humidified atmosphere (5% CO2) in either DMEM (Sigma – Aldrich, UK) (Miapaca-2, Panc-1, HN5 and MCF-7) or RPMI-1640 medium (Sigma – Aldrich, UK) (BxPC3, PT45, AsPc-1, Capan-1 and FA6) supplemented with 10% Foetal Bovine Serum (PAA, UK), antibiotics penicillin (50 units/mL), streptomycin (0.05 mg/mL) and neomycin (0.1 mg/mL) as described previously [19]. RPMI-1640 medium was supplemented with 2mM Glutamine (Sigma - Aldrich, UK).

### Antibodies and other reagents

MAb ICR62 (IgG2b) was raised against the external domain of the EGFR on the breast cancer cell line MDA-MB468 as described previously [21]. The primary mouse anti-IGF-IR antibody used in this study for flow cytometry was purchased from R&D Systems (Abingdon, UK). Secondary FITC-conjugated rabbit anti-mouse mAb STAR9B was obtained from AbD Serotec (Kidlington, UK) while gemcitabine was acquired from Healthcare at Home (UK). PI3K inhibitor LY294002 and MAPKK/MEK inhibitor U0126 were purchased from Cell signaling (UK). The anti-IGF-IR TKI NVP-AEW541 and pan-HER inhibitor afatinib were kindly provided by Novartis (Basel, Switzerland) and Boehringer Ingelheim respectively (Vienna, Austria) [20,22]. Mouse antibodies against HER-2, HER-3, HER-4, p-IGF-IR (Tyr1165/1166) and anti-IGF-IR rabbit antibody were obtained from Santa Cruz, UK. Mouse antibody against β-actin was purchased from Cell Signalling, UK, while mouse anti-EGFR antibody from Sigma-Aldrich, UK. Rabbit antibodies against AKT, MAPK, phospho-MAPK (Thr202/Tyr204), p-HER-3 (Tyr1289), p-HER-2 (Tyr1221/1222) and phospho EGFR (Tyr1086) were purchased from Cell Signalling,UK while anti-phospho AKT (S473) rabbit antibody was obtained from Biosource, UK.

### Determination of cell surface expression of growth factor receptors

The cell surface expression of IGF-IR was assessed by flow cytometry as described previously [19]. Briefly, about 1 million cells were incubated for 1 hour by rotation at 4°C, with the primary antibody or control medium alone. Cancer cells were then washed three times by centrifugation and incubated for 1 hour by rotation at 4°C with FITC-conjugated rabbit anti-mouse IgG STAR9B (AbD Serotec, UK). A minimum of 10.000 events were recorded following excitation with an argon laser at 488 nm using the FL-1 detector (525 nm) of a BD FACsCalibur flow cytometer (Becton Dickinson Ltd, UK). Mean fluorescence intensity values were calculated using the CellQuest Pro software (Becton Dickinson Ltd, UK) and compared with those of negative controls (no primary antibody).

### Cell growth studies

The effect of the various agents, on the growth of human cancer cell lines was investigated using the Sulforhodamine B (SRB; Sigma – Aldrich, UK) colorimetric assay as described previously [19]. Briefly, 5 × 10^3^ tumour cells/well were seeded in 100 μL of growth medium supplemented with 2% FBS in a 96-well plate. After 4 hours incubation at 37°C, 100 μL aliquots of doubling dilutions of the agents were added to triplicate wells. When cells in control wells (no treatment) were almost confluent, cells were fixed with 10% trichloroacetic acid (Fisher Scientific, UK) and stained with 0.4% SRB in 1% acetic acid. SRB stain was solubilised with 10 mM Tris-base (Fisher Scientific, UK) and the absorbance of each well was measured at 565 nm using an Epoch plate reader (Biotek, UK). Growth as a percentage of control was determined as described previously [19]. IC50 values were calculated using the Gen5 software (Biotek, UK).

### Determination of combination index

Interactions between the different agents when used in combination were assessed, using the combination index (CI) as described by Chou and Talalay [23]. For each combination the two drugs were mixed at their 4 × IC_50_ followed by 8 doubling dilutions. CI <0.9 indicates a synergistic effect while CI between 0.90 -1.10 denotes an additive effect. CI >1.1 indicates antagonistic effects. Data analysis was performed using the Calcusyn software (Biosoft, UK).

### Cell cycle distribution analysis

The effect of NVP-AEW541 on the cell cycle distribution of the cancer cell lines was investigated using flow cytometry. Briefly, approximately 2.5 × 10^5^ cells were seeded to 25 cm^2^ flasks containing 10 mL of 2% FBS growth medium and the inhibitors at different concentrations or control medium. Once the cells containing only medium were almost confluent, treated cells were harvested and pooled together with the supernatant and washed three times with cold PBS by centrifugation. The final cell pellet was re-suspended in 200 μL of cold PBS, fixed by the addition of 70% ethanol and incubated overnight at 4°C. Tumour cells were incubated with PI/RNAse mix (Becton Dickinson Ltd, UK) for 35 min at room temperature. A minimum of 10.000 events were recorded by excitation with an argon laser at 488nm using the FL-3 detector (620 nm) of a BD FACsCalibur flow cytometer (Becton Dickinson Ltd, UK) and analysed using the CellQuest Pro software (Becton Dickinson Ltd, UK).

### Western blot analysis

Cancer cells were grown to near confluency in 6-well culture plates containing 5 mL of 10% FBS RPMI growth medium. Cells were washed once with 5 ml of RPMI/0.5% FBS and incubated in 5 mL of RPMI/0.5% FBS containing no inhibitor, NVP-AEW541 (400 nM), afatinib (400 nM) or ICR62 (200 nM) for 24 hours at 37°C. Following incubation with the inhibitors, cells were stimulated with 20 nM of EGF (R&D systems), IGF-I, IGF-II, NRG-1(Cell signaling, UK) or Insulin (Austral Biologicals, California, USA) for 15 min. Cancer cells were lysed using 400 μL of lithium dodecyl sulfate (LDS) lysis buffer (Invitrogen, UK) containing protease inhibitor cocktail (Sigma-Aldrich, UK) and cell lysates were heated at 90°C for 5 min. Protein samples (30 μg) were separated on 4% to 12% Bis-Tris gels (Invitrogen, UK) and transferred to polyvinylidene difluoride (PVDF) membranes (Invitrogen, UK). The PVDF membranes were probed with antibodies at optimal concentrations according to the manufacturer’s instructions. The specific signals were detected using the WesternBreeze chemiluminescence kit (Alkaline phosphatase conjugated secondary antibody) (Invitrogen, UK). Results were visualized using the GenGnome5 imaging system (Syngene, UK).

### Statistical analysis

The unpaired two-tailed Student’s t-test was used for comparing mean values between two groups. Data are presented as mean ± SD. P < 0.05 was considered statistically significant.

## Results

### IGF-IR expression in pancreatic cancer cells

**Figure 1:**
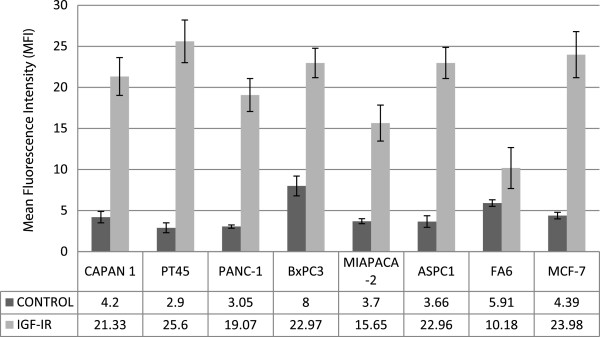
**Expression of IGF-IR in human pancreatic tumour cell lines assessed by Flow Cytometry as described in the Materials and methods.** Results are expressed as Mean Fluorescent Intensity (MFI) values. Breast cancer cell line MCF-7 was used as a positive control.

We have reported recently the cell surface expression levels of HER family members on seven human pancreatic cancer cell lines and found all seven cancer cell lines to be positive for both EGFR and HER-2 , negative for HER-4 while expressing extremely low or undetectable levels of HER-3 [19]. Here, we determined the expression levels of IGF-IR in the same panel of pancreatic cancer cell lines using flow cytometry. All pancreatic tumour cell lines were found to be positive for IGF-IR, with MFIs ranging from 4.2 (FA6) to 22.7 (PT45) (adjusted to negative control) (Figure 1). In the majority of the pancreatic cancer cell lines examined, the IGF-IR expression levels were similar to the IGF-IR expression level in the control MCF-7 breast tumour cell line (MFI = 19.6) (Figure 1).

### Growth response of human pancreatic cancer cell lines to treatment with HER family growth factors, IGF-I, IGF-II and insulin

**Figure 2:**
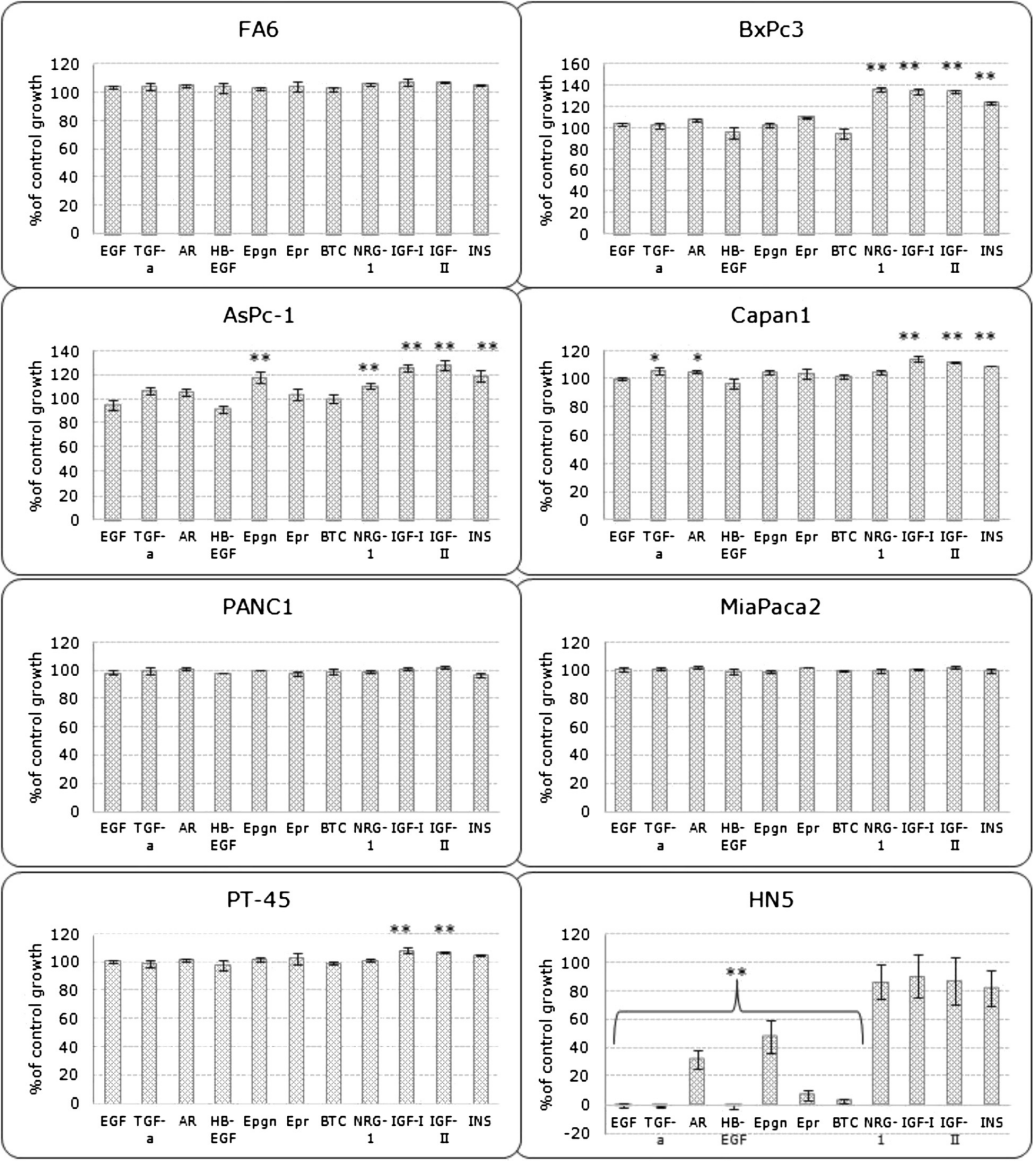
Effect of HER family and IGF-IR growth factors on the growth of human pancreatic cancer cell lines as percentage of control growth (*, p<0.05, **, p<0.01). Cells were treated with 40 nM of EGF, TGFα, AR, Epigen, HB-EGF, Epiregulin, BTC, NRG-1, IGF-I, IGF-II or Insulin for 72 h in growth medium supplemented with 2% FBS. Results are expressed as percentage of control cells (no treatment) calculated as described in the Materials and Methods.

We determined the growth response of human pancreatic cancer cell lines to treatment with EGFR ligands (EGF, TGFα, AR, Epigen), HER-3 and HER-4 ligand NRG-1, EGFR and HER-4 ligands ( HB-EGF, Epiregulin and BTC) , IGF-IR ligands (IGF-I and IGF-II) and insulin at a concentration of 40 nM for 72 h using the SRB assay (Figure 2). For this assay, cells were grown in medium containing 2% FBS as in growth inhibition studies with other agents. We have shown previously that, at nM concentrations, EGFR ligands inhibit the growth of EGFR overexpressing tumour cell lines *in vitro*[24]. To confirm the bioactivity of exogenous HER ligands, we examined their effects on the growth of EGFR overexpressing HN5 cells. All HER ligands, except NRG-1, inhibited the growth of HN5 cells *in vitro* (Figure 2). In addition, with the exception of BxPC3 and AsPc-1 cell lines which exhibited significant growth response to NRG-1 (BxPc3: 36% increase compared to the control, p<0.01, AsPc-1: 19% increase compared to the control, p<0.01), the majority of pancreatic tumour cell lines did not respond to treatment with the exogenous HER ligands or exhibited very low increase in cell proliferation (Figure 2). Interestingly AsPc-1 was the only cell line which exhibited increased growth after treatment with epigen (18.5%, p<0.01). Of all cell lines examined here, only BxPc3,AsPc1, Capan-1 and PT45 cell lines demonstrated significant increase in growth (p<0.01) after treatment with IGF-I, IGF-II or insulin (Figure 2).

### Growth response of human pancreatic tumour cells to treatment with NVP-AEW541 as a single agent or in combination with gemcitabine, afatinib and ICR62

**Figure 3:**
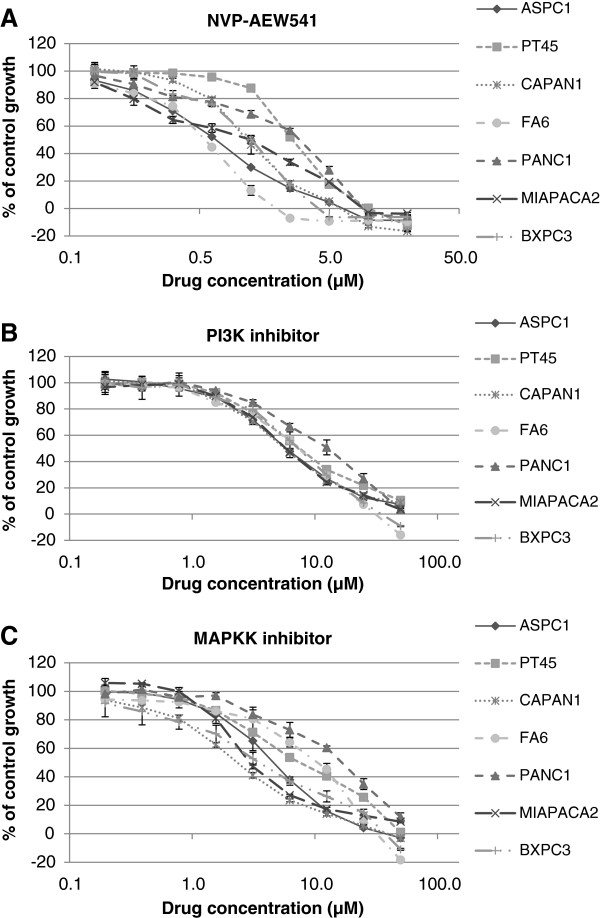
**Effect of doubling dilutions of NVP-AEW541 (A), PI3K inhibitor (B) and MAPK inhibitor (C), on the growth of human pancreatic cancer cell lines.** Tumour cells were grown in the presence of doubling dilutions of the agents or medium alone until control cells (no treatment) were confluent. Cell proliferation was calculated as percentage of control cell growth, as described in the Materials and Methods. Each point represents the mean ±s.d of triplicate samples.

We have reported recently the effect of afatinib, erlotinib, ICR62 and gemcitabine on the growth of pancreatic cancer cell lines [19]. Of these agents gemcitabine exhibited the highest anti-proliferative activity with IC50 values at the low nanomolar range while afatinib with a range of IC50 values from 11nM to 1.37 μM demonstrated a higher anti-tumour activity compared to first generation EGFR TKI erlotinib [19]. Here we investigated the growth response of the same panel of pancreatic cancer cell lines to treatment with NVP-AEW541 an IGF-IR TKI. Of 7 human pancreatic tumour cell lines examined, FA6 cells were the most sensitive cell line to treatment with NVP-AEW541 with an IC50 value of 342 nM (Figure 3, Table 1). The IC50 values for the rest of the cell lines ranged from 897 nM (ASPC1) to 2.73 μM (PT45).

**Table 1:** IC50 values for NVP-AEW541, PI3K and MAPKK inhibitors in pancreatic cancer cell lines as assessed by the SRB colorimetric assay

**ICell line**	**NVPAEW541**	**MAPKK inh**	**PI3K inh**
**BxPc-3**	1.54 (1.37–1.74) μM	3.5 (2.77–4.1) μM	6.9 (6.12–7.96) μM
**AsPc-1**	0.897 (0.79–0.92) μM	4.9 (4.52–5.42) μM	5.5 (5.12–6.06) μM
**FA-6**	0.342 (0.26–0.36) μM	11.5 (10.15–13.08) μM	9.1 (8.53–9.74) μM
**PANC-1**	2.66 (1.8–3.2) μM	13.7 (12.38–15.2) μM	11.3 (10.28–12.59) μM
**Capan-1**	0.969 (0.9–1.04) μM	2.3 (2.23–2.51) μM	5.9 (5.56–6.39) μM
**MiaPaca-2**	1.13 (1.01–1.38) μM	3.5 (3.08–4.05) μM	6 (5.69–6.5) μM
**PT45**	2.73 (2.57–2.87) μM	7.7 (6.97–8.53) μM	8 (7.54–8.52) μM

**Figure 4:**
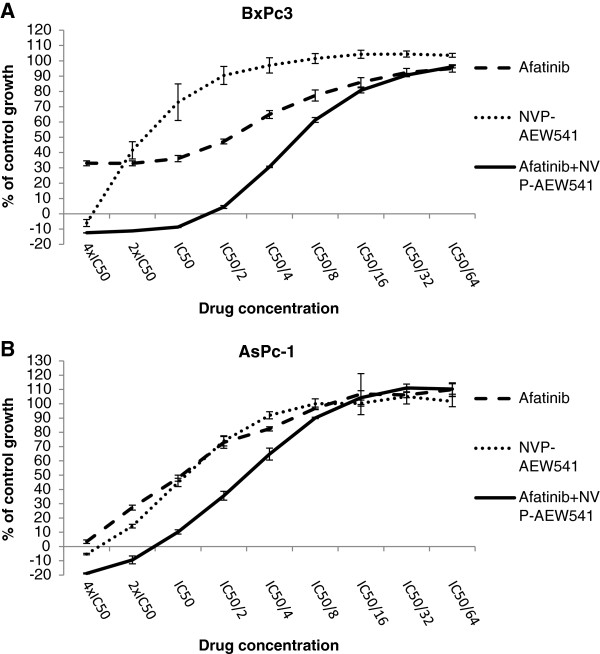
The effect of doubling dilutions (starting at 4xIC50 value followed by 8 doubling dilutions) of the combination of NVP-AEW541 and afatinib compared to single treatment, in (A) BxPc3 , (B) AsPc-1 cell lines.

Median effect analysis showed that a combination of NVP-AEW541 with gemcitabine led to a synergistic or additive growth inhibition of 4 out of 7 human pancreatic tumour cell lines (Table 2). We found no enhancement of growth inhibition following treatment with a combination of ICR62 with NVP-AEW541 (data not shown). Interestingly, with the exception of PT-45, the combination of the IGF-IR inhibitor NVP-AEW541 with afatinib was superior to that of NVP-AEW541 with gemcitabine leading to synergistic growth inhibition of all pancreatic cancer cell lines (Table 2, Figure 4). However, this was statistically significant in four cell lines.

**Table 2:** Mean combination index values of NVP-AEW541 plus gemcitabine or afatinib in pancreatic cancer cell lines (three independent experiments)

**Cell line**	**Mean Combination index (range, effect)**			
	**NVP-AEW541+GEM**	**p-value**	**NVP-AEW541+Afatinib**	**p-value**
BxPc-3	0.96 (0.92–1.08, Additive)	p=0.54	0.34 (0.29–0.44, Synergism)	p<0.05
AsPc-1	0.91 (0.86–0.95, Additive)	p<0.05	0.75 (0.68–0.84, Moderate Synergism)	p<0.05
FA-6	1.22 (1.07–1.33, Moderate antagonism)	p<0.05	0.8 (0.68–0.94, Moderate Synergism)	p=0.057
PANC-1	0.7 (0.56–0.84, Synergism)	p<0.05	0.73 (0.61–0.86, Moderate Synergism)	p<0.05
Capan-1	1.43 (1.31–1.52, Moderate antagonism)	p<0.05	0.9 (0.81–1.05, Slight Synergism/Additive)	p=0.34
MiaPaca-2	1.14 (1.02–1.27, Slight antagonism)	p=0.11	0.84 (0.78–0.91, Moderate Synergism)	p<0.05
PT45	1.09 (0.92–1.23, Additive)	p=0.36	1.44 (Moderate antagonism)	p<0.05

Interpretation of the results was based on the proposed descriptions for presenting the degrees of antagonism or synergism by Calcusyn software. P values indicate level of statistical significance compared with a combination index value of 1.

In order to investigate the response of the pancreatic cancer cell lines to direct inhibition of RAS/RAF/MAPK and PI3K/AKT signalling cascades as well as their dependency on these pathways, we determined the growth response of these cell lines to treatment with the PI3K inhibitor LY294002 and MAPKK/MEk inhibitor U0126. Both inhibitors were found to be less effective at inhibiting the growth of pancreatic cancer cell lines compared to IGF-IR inhibitor NVP-AEW541, with IC50s ranging from 2.3 μM (Capan-1) to 13.7 μM (PANC1) for MAPKK inhibitor and 5.5 μM (AsPc-1) to 11.3 μM (PANC1) for the PI3K inhibitor (Table 1). Interestingly, the most resistant cell lines to PI3K inhibition were also found to be resistant to anti-MAPKK treatment (Table 1, Figure 3B,C).

### Cell-cycle distribution analyses

We used flow cytometry in order to determine the effect of NVP-AEW541 (IC70 concentration) on the cell cycle distribution of the pancreatic cancer cell lines. We have reported recently that treatment with gemcitabine increased the percentage of cells in the sub-G1 and S phase while afatinib increased the proportion of cells in the sub-G1 and this was accompanied by a decrease in the population of cells in G0/G1 [19]. Similarly, an increase in the sub-G1 fraction, indicative of apoptosis, was observed in the majority of cell lines following NVP-AEW541 treatment and this was statistically significant in FA6, AsPC-1, PT45 and Capan-1 cells (Table 3). An increase in the percentage of cells in G0/G1 phase was demonstrated only in five out of the seven cell lines and this increase was statistically significant in BxPc3 and PANC1 (Table 3).

**Table 3:** Effect of NVP-AEW541 (IC70) on the cell cycle distribution of pancreatic cancer cell lines

**Cell line/treatment**	**Sub-G1**	**G0/G1**	**S**	**G2/M**
*BxPc3*				
Control	7.7±1.4	55.6±2.3	25.4±5	10.8±1.8
NVP-AEW541	7.9±2.7	69.7±4.5*	15.7±2.9	6.2±1.1
*AsPC-1*				
Control	1±0.02	46.1±0.3	31.8±0.2	19.5±0.1
NVP-AEW541	3.9±0.2*	47.1±0.4	35.7±1.5	14±2.6
*Capan-1*				
Control	3.8±0.3	50.6±2.5	25.5±3.1	18.6±1.1
NVP-AEW541	7±0.2*	53.6±1.3	23±1.8	15.1±1.4
*PT45*				
Control	2.3±0.6	74.4±4.3	12.3±2.8	10.1±1
NVP-AEW541	10.2±2.7*	59.8±1.4*	19.7±4.8	9.9±2.4
*Miapaca-2*				
Control	4.6±2.6	76.1±2.2	10±1.3	8.6±0.5
NVP-AEW541	5.5±2.7	81±1.3	9.4±0.8	4.5±3.7
*PANC1*				
Control	5.6±1.5	48.3±4.6	11.6±0.1	32.7±4.7
NVP-AEW541	9.3±0.5	72.4±2.1*	12.1±0.3	4.9±0.4*
*FA6*				
Control	13.8±2	48.3±3.1	25.4±4.8	11.2±0.4
NVP-AEW541	36.8±2.8*	33.2±1.2*	22.2±1.3	6.9±2.2

Each population is expressed as percentage of gated cells (mean of three independent experiments ± S.D).* depicts statistically significant difference (p<0.05) compared to control values.

### Effect of HER and IGF-IR ligands in the presence or absence of inhibitors on downstream cell signaling molecules

**Figure 5:**
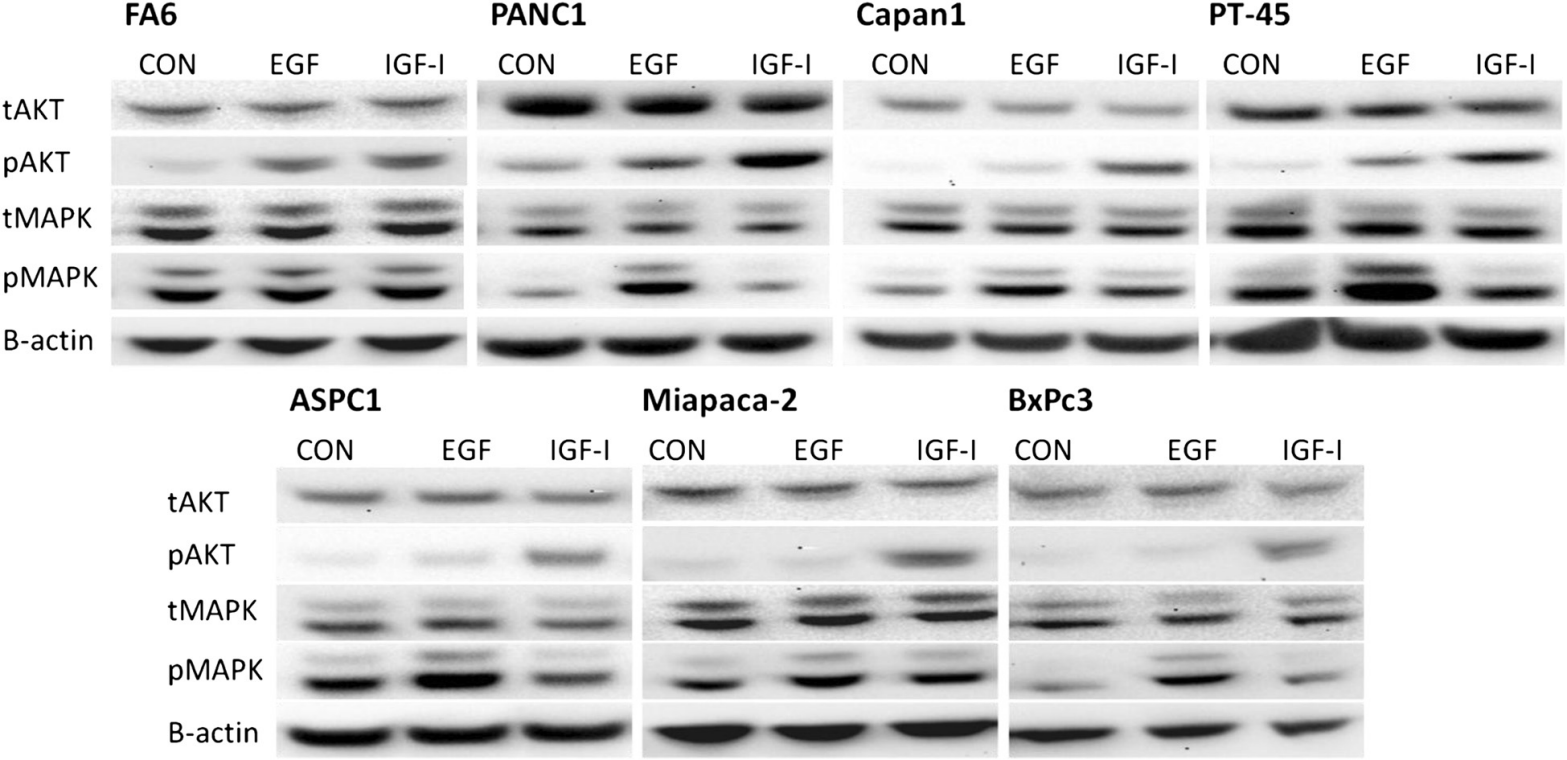
**Effect of IGF-I and EGF 20 nM (for 15 min) on downstream signaling pathways in all pancreatic cancer cell lines used in this study.** Cells were grown to near-confluency in 10% FBS growth followed by 24 h incubation in 0.1% FBS growth medium at 37°C. Following that, cells were stimulated with 20 nM of EGF and IGF-I for 15 min. Cells were lysed, protein samples were separated by SDS-PAGE, transferred onto PVDF membranes and probed with the antibodies of interest.

First we determined the effect of EGF and IGF-I on the phosphorylation of AKT and MAPK in all pancreatic cancer cell lines included in this study and in all cell lines, with the exception of FA6 cells, EGF primarily induced to the activation of MAPK while it had low or no effect on AKT phosphorylation. In contrast, IGF-I was more potent in inducing the activation of AKT, while having no or minimal effect on MAPK phosphorylation (Figure 5).

**Figure 6:**
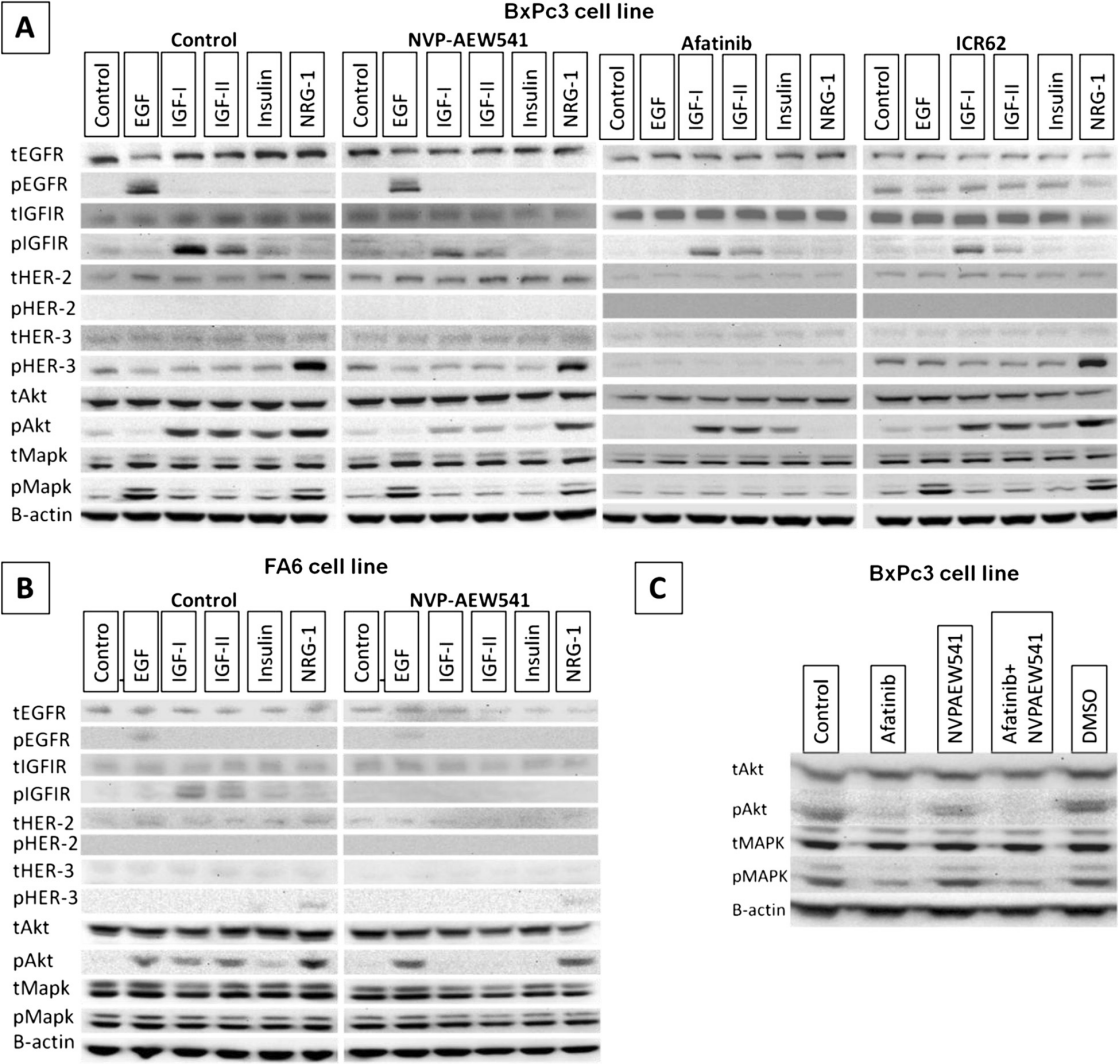
**Effect of IGF-I, IGF-II, Insulin, EGF and NRG-1 at in the presence or absence of IGF-IR and/or HER inhibitors (400 nM) in BxPc3 (A) or FA6 (B) cell line (overnight starved).** Cells were grown to near-confluency in 10% FBS growth medium, then treated with the inhibitors in 0.1% FBS growth medium at 37°C. Following 24 h incubation with the inhibitors or growth medium alone cells were stimulated with 20 nM of various growth factors for 15 min. Cells were lysed , protein samples were separated by SDS-PAGE, transferred onto PVDF membranes and probed with the antibodies of interest. **C**). Effect of afatinib (400 nM) and NVPAEW541 (400 nM) when used alone or in combination in BxPc3 cells in 10% FBS growth medium.

Next, we examined the effect of EGF, IGF-I, IGF-II, insulin and NRG1 on the activation of downstream signaling pathways in BxPc3 cell line in the presence or absence of afatinib, NVP-AEW541 or mAb ICR62 (Figure 6A). BxPc3 cell line was selected as the most appropriate model for investigating cell signaling events since the combination of afatinib with NVP-AEW541 exhibited the highest synergistic effect in these cells (lower CI value) (Table 2). In addition, BxPc3 cell line was positive for all HER family members and IGF-IR with the exception of HER-4 [19].

Of the HER ligands, EGF induced phophorylation of EGFR and MAPK while NRG1 induced phosphorylation of HER-3 and both of MAPK and AKT in BxPC-3 cells and these effects were blocked completely by afatinib (Figure 6A, afatinib). In addition, treatment with IGF-IR ligands increased the level of p-IGF-IR and pAKT but not pMAPK. At 400 nM NVP-AEW541 inhibited the IGF-IR ligands induced phosphorylation of both IGF-IR and AKT but not completely (Figure 6A, NVP-AEW541).

Next we investigated the effect of the above mentioned ligands in downstream signaling in the presence or absence of NVP-AEW541 in FA6 cells which was the most sensitive cell line to treatment with this agent. Interestingly, in contrast to BxPc3 cells, NVP-AEW541 (at 400 nM) inhibited completely the ligand-induced phosphorylation of IGF-IR and Akt. The basal levels of pMAPK were found to be higher in the FA6 cell line compared to BxPC3 cells and this was not increased further following treatment with IGF-IR or HER ligands (Figure 6B).

Finally, we determined whether afatinib and NVP-AEW541, when used alone or in combination, have the same effects in BxPc3 cells grown at optimal conditions (i.e. medium containing 10% FBS). Only afatinib downregulated the basal levels of pMAPK . In addition, it was also more potent compared to NVP-AEW541 at downregulating of pAKT. However, only the combination of these two inhibitors (i.e. afatinib plus NVP-AEW541) led to complete downregulation of the pAKT basal levels (Figure 6C).

## Discussion

Despite significant advances in the understanding of cancer biology during recent decades, pancreatic cancer remains one of the deadliest types of human cancer [1-3]. Since the introduction of gemcitabine in 1996, which is currently the gold standard for the treatment of advanced pancreatic cancer, only the EGFR TKI erlotinib has gained FDA approval for the treatment of patients with metastatic pancreatic cancer in combination with gemcitabine [25]. This combination resulted in a modest, but statistically significant survival benefit however, many patients simply do not respond or acquire resistance following a short course of therapy [25,26]. Recent studies have demonstrated that IGF-IR is implicated in resistance to anti-HER targeted therapy and that simultaneous targeting of both IGF-IR and EGFR or IGF-IR and HER-2 may lead to a superior therapeutic effect compared to treatment with the single agent in breast and glioblastoma, prostate and colorectal cancer cells [27-36].

To date, the number of studies investigating the effect of IGF-IR inhibitor NVP-AEW541, in pancreatic cancer is limited [37-39]. To the best of our knowledge this is the first study investigating the therapeutic potential of this approach in pancreatic cancer using a pan-HER bocker (afatinib) and IGF-IR TKI NVP-AEW541. We have reported recently the superiority of afatinib compared to our anti-EGFR mAb ICR62 and erlotinib in inhibiting the growth of a panel of human pancreatic cancer cell lines. As a single agent, afatinib inhibited the growth of all pancreatic cancer cell lines with IC50 values ranging from 11 nM (BxPC-3) to 1.37 μM (FA6) [19]. Interestingly, BxPC-3, which is the only one carrying a wild-type K-Ras gene, was the most sensitive cell line to treatment with HER inhibitors [19]. In addition, we found that treatment with a combination of afatinib and gemcitabine resulted in the synergistic growth inhibition of the majority of human pancreatic cancer cells (BxPC-3, AsPc-1, FA6, PANC-1 and Capan-1) [19]. In this study, we investigated the sensitivity of the same panel of pancreatic cancer cells to treatment with NVP-AEW541 when used alone or in combination with gemcitabine, ICR62 or afatinib. We found NVP-AEW541 to inhibit the growth of all pancreatic cancer cell lines with IC50 values ranging from 342 nM (FA6) to 2.73 μM (PT-45) (Figure 3, Table 1). Western blot analysis revealed that, NVP-AEW541 inhibited completely the ligand-induced phosphorylation of IGF-IR and AKT in FA6 but not in the more resistant BxPC3 cells (IC50= 1.54 μM) (Table 1, Figure 6). We also investigated the growth response of these cancer cell lines to treatment with PI3K and MAPKK inhibitors and found that these were less effective compared to afatinib and NVP-AEW541 (Figure 3, Table 1). Since the IC50 values of these inhibitors for their respective targets are below 2 μM (0.07 μM for MAPKK inhibitor, 1.4 μM for PI3K inhibitor), our results suggest that the panel of pancreatic cancer cell lines used in this study is highly resistant to inhibition of PI3K and MAPKK.

We next assessed the anti-tumour activity of these agents when used in combination. There was no improvement in anti-tumour activity when NVP-AEW541 was used in combination with mAb ICR62 (data not shown). Treatment with a combination of gemcitabine and NVP-AEW541 resulted in synergistic growth inhibition only in PANC1 cell line (Table 2). Interestingly, treatment with a combination of NVP-AEW541 and afatinib was found to be superior, leading to a synergistic growth inhibition of all pancreatic cancer cells with the exception of PT45 which was the most resistance cell line to treatment with NVP-AEW541 (Table 2). Synergism following treatment with a combination of NVP-AEW541 and HER inhibitors (e.g. trastuzumab, erlotinib) has previously been reported in studies involving breast and colorectal cancer cells [36,40,41].

Investigation of the effect of IGF-IR ligands (IGF-I, IGF-II and Insulin) and HER ligands EGF and NRG-1 on the downstream signaling in BxPc3 cells revealed that EGF primarily induces phosphorylation of MAPK while IGF-IR ligands activate predominantly the PI3K/AKT pathway. The activation of different pathways by the HER family and IGF-IR systems could explain the synergistic effect exhibited by the combination of pan-HER blocker afatinib and IGF-IR inhibitor in this cell line. In optimal growth conditions (10% FBS supplemented medium) afatinib was more potent at down regulating both AKT and MAPK basal phosphorylation levels while NVP-AEW541 downregulated pAKT but had no effect on pMAPK basal levels in BxPc3 cells. However, even though afatinib was more effective at downregulating pAKT than NVP-AEW541, only the combination of NVP-AEW541 with afatinib led to complete loss of AKT phosphorylation (Figure 6C).

In order to determine whether the diverse activation of AKT and MAPK pathways by EGFR and IGF-IR activation could explain the synergism exhibited by the same combination in the rest of the cell lines we determined the effect of EGF and IGF on the phosphorylation of AKT and MAPK in all cell lines included in this study. Interestingly, with the exception of FA6 cells, the pattern of AKT and MAPK activation in all the other pancreatic cells was found to be similar to BxPc3 cells (Figure 5); EGF predominantly led to the activation of MAPK whereas IGF treatment increased mainly the phosphorylation of AKT but had low or no effect on phosphorylation of MAPK. This in turn suggests that the synergistic effect by this combination may be driven by more effective and simultaneous blockade of HER family members and IGF-IR induced phosphorylation of both AKT and MAPK. However, further studies investigating the effect of this combination in other signaling pathways such as the JAK-STAT pathway, and the effect of the mutational status of downstream cell signalling molecules (e.g. IRS, PTEN and K-ras), on the synergistic potential of this combination, are necessary in order to elucidate the exact mechanism involved in the synergism observed by this combination.

All of the pancreatic cancer cell lines examined in this study were found to be IGF-IR positive, and in the majority of the cases, the expression levels were similar to that of the IGF-IR positive MCF-7 control cell line consequently, there was no correlation between IGF-IR expression and response to treatment with NVP-AEW541, indicating that additional factors are implicated in the sensitivity of these cell lines to IGF-IR inhibition (Table 1). Lack of any clear association between IGF-IR expression and response to NVP-AEW541 has also been found in previous studies investigating the effect of this agent against colorectal and breast cancer cell lines [35,42].

In order to investigate the dependency of each cell line to the HER and IGF-IR signalling pathways, we determined the growth response of these cell lines to several exogenous HER and IGF-IR ligands. Results showed that while the majority of cell lines did not respond to treatment with exogenous HER ligands, several cell lines demonstrated increased growth following treatment with IGF-IR ligands (BxPc3, AsPc-1, Capan-1 and PT45) indicating that IGF-IR may have a more important biological role in this panel of pancreatic cancer cell lines. In addition, the fact that two cell lines (AsPc-1 and Capan-1) responded to some HER ligands but not others (e.g. AsPc-1 responded to epigen treatment only but not to any other EGFR ligand) indicates that different ligands can have a diverse impact on the proliferation of each pancreatic cancer cell line (Figure 2). Furthermore, our results suggest that there is no correlation between growth response to these exogenous ligands and inhibition of their respective receptors. For example, FA6 cell line which exhibited the highest sensitivity to IGF-IR inhibition (IC50 = 342 nM) by TKI NVP-AEW541, was growth stimulated by 5-7% following treatment with either IGF-I, IGF-II or insulin. In contrast, BxPc3, which is a more resistant cell line to IGF-IR inhibition (IC50 = 1.54 μM), exhibited more than 30% increase in growth following treatment with the same ligands (Figure 2). Therefore, other factors such as the level of autocrine ligands, the expression and status of downstream cell signalling molecules, as well as the level of cross-talk between different RTKs may influence sensitivity to IGF-IR inhibition [8,43].

Several studies investigating the therapeutic potential of IGF-IR inhibition have been met with disappointing results, indicating that the potential of this receptor as a single target may be rather limited [44]. Interestingly, our results show that NVP-AEW541 is effective at inhibiting the growth of human pancreatic tumour cells and that the combination of NVP-AEW541 and afatinib is superior in terms of synergistic effect to the combination of either agent with gemcitabine. Taken together, our findings encourage further investigation *in vivo* on the therapeutic potential of this combination in pancreatic cancer.

## Conclusion

Our results indicate that co-targeting of the erbB (HER) family and IGF-IR, with a combination of afatinib and NVP-AEW541, is superior to treatment with a single agent and encourages further investigation on their therapeutic potential in IGF-IR and HER positive pancreatic cancers.

## Competing interest

Professor Helmout Modjtahedi received funding from Boehringer Ingelheim towards conference expenses for his PhD students. We have no Financial or non-financial competing interests.

## Authors’ contribution

NI carried out all these experiments as part of his PhD under the supervision of HM (Director of studies) and his other PhD supervisors AD, AS and DM. All authors read and approved the final manuscript.

## Pre-publication history

The pre-publication history for this paper can be accessed here:

http://www.biomedcentral.com/1471-2407/13/41/prepub
